# Changes in peripheral blood immune cell population in thyroid cancer patients treated with lenvatinib

**DOI:** 10.1038/s41598-023-39503-w

**Published:** 2023-08-07

**Authors:** Meihua Jin, Chae A. Kim, Dong Jun Bae, Sang-Yeob Kim, Tae Yong Kim, Won Bae Kim, Young Kee Shong, Won Gu Kim, Min Ji Jeon

**Affiliations:** 1 Division of Endocrinology and Metabolism, Department of Internal Medicine, Asan Medical Center, University of Ulsan College of Medicine, 88, Olympic-ro 43-gil, Songpa-gu, Seoul, 05505 Republic of Korea; 2 PrismCDX Co., Ltd., 593-16, Dongtan Giheung-ro, Hwaseoung-si, 18469 Gyeonggi-do Korea; 3 Convergence Medicine Research Center, Asan Institute for Life Sciences, Asan Medical Center, Seoul, 05505 Korea; 4 Division of Endocrinology and Metabolism, Dankook University College of Medicine, Cheonan, 3116 Korea

**Keywords:** Cancer, Endocrinology

## Abstract

This study evaluated changes in the peripheral blood immune cell population in patients with advanced thyroid cancer receiving lenvatinib treatment to confirm the immune-modulatory effect of lenvatinib. After obtaining informed consent from patients, we prospectively collected 20 ml of whole blood at 2–3 months intervals 2–4 times from each patient; peripheral blood mononuclear cells (PBMCs) were separated, and the Maxpar Direct Immune Profiling Assay was performed. A total of 10 patients were enrolled, and 31 blood samples were obtained. The median age of patients was 65 years, and all patients showed durable responses to the lenvatinib treatment. When we compared the PBMC profiles between the pre-treatment, on-treatment, and off-treatment samples, the peripheral natural killer (NK) cell proportion differed significantly. The proportion of NK cells among total live cells significantly increased from 9.3 ± 4.5 (%) in the pre-treatment samples to 20.8 ± 7.9 (%) in the on-treatment samples (*P* = 0.009) and decreased to 13.3 ± 3.1 (%) in the off-treatment samples (*P* = 0.07). There was a significant increase in the peripheral NK cell population with lenvatinib treatment in advanced thyroid cancer patients. This finding confirms the immune-modulatory effect of lenvatinib.

## Introduction

Lenvatinib is a multitargeted tyrosine kinase inhibitor of vascular endothelial growth factor receptor (VEGFR) 1–3, fibroblast growth factor receptor (FGFR) 1–4, platelet-derived growth factor receptor alpha, ret proto-oncogene, and stem cell factor receptor^1,2^. Therefore, the main efficacy of lenvatinib against cancer is associated with its antiangiogenic and antiproliferative effect^2–4^. Both sorafenib and lenvatinib have been used to treat radioactive iodine (RAI)-refractory differentiated thyroid carcinoma (DTC), but lenvatinib showed a better efficacy than sorafenib in clinical trials and a recent real-world study^5–7^.

There are several possible mechanisms that can explain the superior efficacy of lenvatinib. First, lenvatinib exerts an additional inhibitory effect through blocking FGFR compared to sorafenib. The FGFR pathway offers an intracellular alternative to the VEGFR pathway, thus preventing the development of resistance to VEGF/VEGFR inhibitors^8–10^. Another mechanism is the immunomodulatory effect of lenvatinib. Several preclinical studies have demonstrated its action on the tumor microenvironment via enhancing tumor infiltration, activating natural killer (NK) cells, and decreasing the number of tumor-associated macrophages^2,11–13^. However, these actions have not been confirmed in clinical studies. Investigating the changes in tumor-infiltrating immune cells during lenvatinib treatment is challenging. As an alternative, peripheral immune cells can be used to reflect host immunity^14–16^.

In this study, we aimed to confirm the immune-modulatory activities of lenvatinib in patients with advanced thyroid cancer. We evaluated changes in the peripheral blood immune cell population and lymphocyte-to-monocyte ratio (LMR) due to lenvatinib treatment. We also investigated the changes in cytokines associated with peripheral blood immune cell changes.

## Materials and methods

### Patients

This prospective cohort study enrolled patients with RAI-refractory, advanced thyroid cancer taking lenvatinib or planning to take lenvatinib from Feb 2020 to Jul 2021. Twenty milliliters of whole blood was sampled from each patient every 2–3 months, approximately 3–4 times per patient. All patients gave their informed consent for inclusion before they participated in this study. The study was conducted in accordance with the Declaration of Helsinki and was approved by the Institutional Review Board of Asan Medical Center (No. 2019-1131). Initial tumor staging was evaluated based on the 8th edition of the tumor-node-metastasis (TNM-8) staging system and disease response was evaluated based on the Response Evaluation Criteria in Solid tumors (RECIST) version 1.1.

### Mass cytometry

After blood sampling, whole blood samples were immediately processed to separate peripheral blood mononuclear cells (PBMCs) and plasma. Briefly, the blood samples were diluted to a 1:1 ratio with wash buffer and added to the Lymphoprep™ solution (Stemcell technologies, Cat 7851, Vancouver, BC, Canada). After centrifuging at 1000*g* at room temperature for 10 min, we collected the plasma and cells in a buffy coat layer. After centrifuging the cells a second time at 450*g* for 15 min and two washing steps, PBMCs were aliquoted. PBMCs and plasma were stored at − 80 °C and preserved viable for further analysis.

PBMCs were stained using the Maxpar Direct Immune Profiling Assay kit (Cat. No. 201325, Fluidigm Corporation, San Francisco, CA, USA) on Helios, a CyTOF system (Fluidigm), according to the manufacturer’s instructions (Supplementary Table 1). Briefly, aliquots of 3 × 10^6^ PBMCs were prepared with 25 µl of Maxpar Cell Staining Buffer (Fluidigm). For Fc-receptor blocking, Human TruStain FcX (BioLegend, San Diego, CA, USA) was added to each tube and incubated for 10 min at room temperature. Fc-receptor blocked PBMCs were directly transferred into a 5 ml tube containing the dry antibody pellet (Fluidigm) and incubated for 30 min at room temperature. After washing, PBMCs were fixed with 1.6% formalin for 10 min at room temperature. Finally, fixed PBMCs were incubated with Cell-ID Intercalator-Ir (Fluidigm) in Maxpar Fix and Perm Buffer (Fluidigm) for up to 48 h at 4 °C. Prior to the acquisition, PBMCs were washed with Maxpar Cell Staining Buffer (Fluidigm) twice and filtered through 40-µm cell strainers before being acquired on a Helios mass cytometer (Fluidigm). Mass cytometry data files were analyzed using FCS Express 7 Flow software (Cat. No. 402001, De Novo Software, CA, USA). Phenotype definitions of cell populations were listed in Supplementary Table 2. The proportion of PBMCs was visualized by the t-Stochastic Neighbor Embedding (t-SNE) plotting using Cytobank (Beckman Coulter, IN, USA).

### Cytokine assay

Plasma levels of IL-2, IL-10, IL-15, and TGFβ were measured using Simoa reagent kits (Quanterix, MA, USA) on the Simoa HD-1 Analyzer (Quanterix, MA, USA) at PrismCDX (Gyeonggi-do, South Korea) by an investigator who was blinded to the clinical information. Eighty microliters of plasma was diluted 1:4 for IL-2, IL-10, and IL-15 or 1:16 for TGFβ according to the Quanterix guidelines. The beads were pre-coated with each capture antibody and added to a disposable cuvette. After bead collection via magnetic separation, either a protein standard or plasma sample was added. The protein standard or sample was incubated with the beads for a total of 20 (IL-10 and TGFβ) or 40 (IL-2 and IL-15) min. Then, the beads were washed and incubated with the biotinylated detector antibody for 7 min. After washing, streptavidin β-galactosidase was added to the beads and incubated for 7 min. Finally, the enzyme substrate (resorufin β-d-galactopyranoside) was added to the beads, and the mixture was loaded onto a disc containing an array of 216,000 microwells and sealed with oil. Both fluorescence and white light images were taken of each well. The average number of enzymes per bead was calculated from the fraction of active wells. All samples were assayed in duplicate, and the mean concentration of analyte with their coefficient variation was calculated.

### Statistical analysis

The data were analyzed using the software R, version 3.4.4 (R Foundation for Statistical Computing; www.R-project.org), and graphs were plotted using GraphPad Prism version 8.0 (GraphPad Software, Inc., San Diego, CA). Continuous variables are presented as the median and interquartile range (IQR) or mean with standard deviation (SD). Categorical variables are presented as a number (percentage). Student’s *t*-test was used to compare the continuous variables. All *P* values less than 0.05 were considered statistically significant.

## Results

### Baseline characteristics of enrolled patients

**Figure 1:**
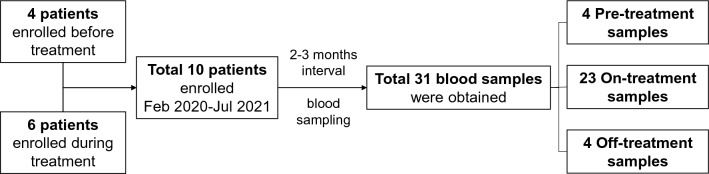
Flowchart of the study.

A total of 10 patients were enrolled, and a total of 31 blood samples were obtained (Fig. 1). Four samples from 4 patients were obtained before the initiation of lenvatinib treatment. Six patients were enrolled while taking lenvatinib, and the median duration between the initiation of lenvatinib treatment to the first blood sampling was 27 (IQR 23–32) months. While taking lenvatinib, all patients underwent blood sampling every 2–3 months (median 2.7 [IQR 2.3–3.0]), and 23 on-treatment samples were obtained. The other four samples were obtained during the drug interruption period because of the side effects of lenvatinib. The median duration between the last dose of lenvatinib and the blood sampling was 49 (IQR 13–98) days.

The baseline clinical characteristics of the patients are described in Table 1. The median age was 64.5 years, and four (40%) patients were male. Six patients were diagnosed with papillary thyroid carcinoma, and four patients each were diagnosed with follicular thyroid carcinoma and poorly differentiated carcinoma. Based on the 8th TNM staging system, 6 (60%) patients were initially classified into stage IV, and the median duration between initial thyroid cancer diagnosis to lenvatinib treatment was 5.0 (IQR 2.5–6.8) years. Lenvatinib was used as the first line therapy in 8 patients (Patient No 1–8), and as salvage therapy followed by sorafenib in 2 patients (Patient No. 9 and 10). These two patients had their first blood collection 33 months and 45 months after switching from sorafenib to lenvatinib, respectively. The median maintenance dose of lenvatinib was 19 (IQR 14–20) mg. All the patients showed good durable responses to lenvatinib treatment by the end of study; two patients with a partial response (Patient No. 2 and 4) as the best response, and the other 8 with stable disease.

**Table 1:** Baseline characteristics of enrolled patients.

	Total (N = 10)
Age (years)	64.5 (60.3–68.5)
Sex, male	4 (40%)
Pathology	
PTC	6 (60%)
FTC	2 (20%)
PDTC	2 (20%)
Initial cancer stage^a^	
I	2 (22%)
II	1 (11%)
IV	6 (67%)
Cumulative RAI dose (mCi)	240 (163–519)
Previous sorafenib treatment (yes)	2 (20%)
Duration between initial diagnosis to lenvatinib treatment (years)	5.0 (2.5–6.8)
Target lesions	
Lymph node	5 (50%)
Lung	4 (40%)
Bone	1 (20%)
Lenvatinib maintenance dose (mg)	19 (14–20)
Best response from lenvatinib	
Partial response	2 (20%)
Stable disease	8 (80%)

Continuous variables are presented as median (interquartile range) and categorical variables as numbers (percentages).

*PTC* papillary thyroid carcinoma, *FTC* follicular thyroid carcinoma, *PDTC* poor differentiated thyroid carcinoma, *RAI* radioactive iodine.

^a^Initial stage by 8th TNM staging system could be assessed in 9 patients.

### Changes in PBMC profiles according to lenvatinib treatment

The PBMC profiles of total samples are summarized in Table 2. The proportion of viable cells was 76.1 ± 7.9 (%), and the proportion of each cell type of total live cells was presented. Total lymphocytes accounted for 56.6 ± 14.7 (%) of total live cells, including 12.3 ± 6.3 (%) of CD8+ T cells and 14.9 ± 7.0 (%) of CD4+ T cells. When we compared the PBMC profiles between the pre-treatment, on-treatment, and off-treatment samples, only the peripheral NK cell proportion differed significantly. The proportion of NK cells among total live cells significantly increased from 9.3 ± 4.5 (%) to 20.8 ± 7.9 (%) after the lenvatinib treatment (*P* = 0.009). This trend was observed both for early and late NK cell subpopulations (*P* = 0.004 and *P* = 0.02, respectively). The proportion of total NK cells decreased from 20.8 ± 7.9 (%) in on-treatment samples to 13.3 ± 3.1 (%) in the off-treatment samples (*P* = 0.07). The decrease in early NK cells in off-treatment samples was also statistically significant (*P* = 0.02). Supplementary Table 3 shows the absolute number of total, early, and late NK cells. The number of total, early, and late NK cells was significantly increased after lenvatinib treatment (*P* < 0.001). Early NK cell count was significantly decreased after discontinuation of lenvatinib treatment (*P* = 0.02). The proportion of total NK cells in CD45+ cells was also significantly increased from 9.7 to 19.2% after lenvatnib treatment (*P* = 0.02, Supplementary Table 3).

**Table 2:** Peripheral blood immune cell population according to lenvatinib treatment.

	Cell proportion of total live cells (%)				*P* value^a^	*P* value^b^
	Total	Pre-treatment (4 samples from 4 patients)	On-treatment (23 samples from 10 patients)	Off-treatment (4 samples from 4 patients)		
Total lymphocytes	56.5 (14.7)	47.4 (24.3)	57.7 (12.9)	58.9 (15.2)	0.21	0.87
CD8+ T cells	12.3 (6.3)	10.9 (7.0)	12.2 (6.2)	14.3 (7.0)	0.71	0.54
Central memory	0.9 (0.5)	1.2 (0.7)	0.8 (0.5)	0.9 (0.3)	0.29	0.95
Effector memory	1.9 (1.1)	1.4 (1.1)	1.9 (1.1)	2.4 (1.1)	0.47	0.32
Terminal effector	7.5 (5.1)	7.1 (4.6)	7.3 (5.3)	9.6 (5.0)	0.95	0.42
CD4+ T cells	14.9 (7.0)	17.0 (8.8)	14.0 (6.4)	18.2 (9.2)	0.41	0.26
Central memory	7.1 (4.2)	9.7 (4.6)	6.5 (3.8)	8.1 (5.5)	0.13	0.46
Effector memory	1.9 (1.5)	1.8 (2.3)	1.7 (1.3)	3.2 (1.7)	0.86	0.06
Terminal effector	3.0 (1.9)	3.8 (2.3)	2.6 (1.7)	4.5 (2.0)	0.24	0.06
Regulatory T cells	0.2 (0.1)	0.2 (0.2)	0.1 (0.1)	0.2 (0.1)	0.49	0.21
B cells	8.6 (4.5)	8.8 (5.9)	8.1 (4.1)	11.3 (5.4)	0.78	0.18
NK cells	18.3 (8.2)	9.3 (4.5)	20.8 (7.9)	13.3 (3.1)	0.009	0.07
Early	2.8 (1.0)	1.6 (0.3)	3.1 (0.9)	1.9 (0.4)	0.004	0.02
Late	15.6 (7.5)	7.7 (4.6)	17.6 (7.4)	11.4 (2.8)	0.02	0.11
Monocytes	18.3 (8.3)	25.3 (12.5)	16.9 (6.2)	19.4 (13.0)	0.27	0.73
Dendritic cells	0.9 (0.5)	1.1 (0.6)	0.9 (0.5)	1.1 (0.7)	0.34	0.54
LMR	4.0 (2.4)	2.6 (2.2)	4.1 (2.2)	4.7 (3.6)	0.23	0.65

Continuous variables are presented as means (standard deviations).

*LMR* lymphocyte to monocyte ratio.

^a^*P* values for the comparison between the pre-treatment (4 samples from 4 patients) and on-treatment (23 samples from 10 patients).

^b^*P* values for the comparison between the on-treatment (23 samples from 10 patients) and off-treatment (4 samples from 4 patients).

Monocytes accounted for 18.3 ± 8.3 (%) of total live cells, and the LMR was 4.0 ± 2.4 in the total samples. When we compared the pre-treatment and on-treatment samples, we found decrease in monocytes and increase in LMR with the lenvatinib treatment, but none were statistically significant.

### NK cell changes according to lenvatinib treatment

**Figure 2:**
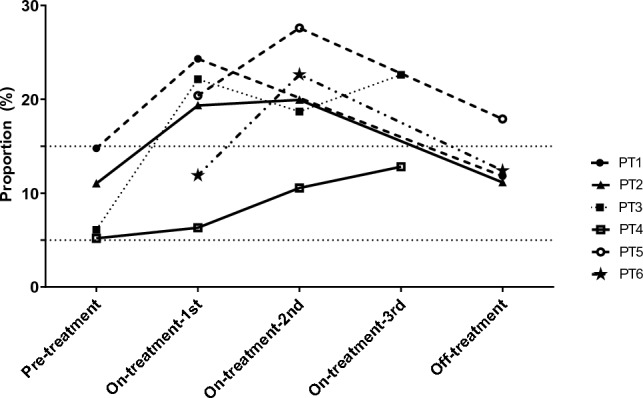
Changes in natural killer (NK) cell proportion (% of total live cells) during lenvatinib treatment in 6 patients with either pre-treatment or off-treatment samples. Four patients with only the on-treatment blood samples were excluded from this figure. On-treatment samples were obtained 2–3 months interval. PT, patient.

**Figure 3:**
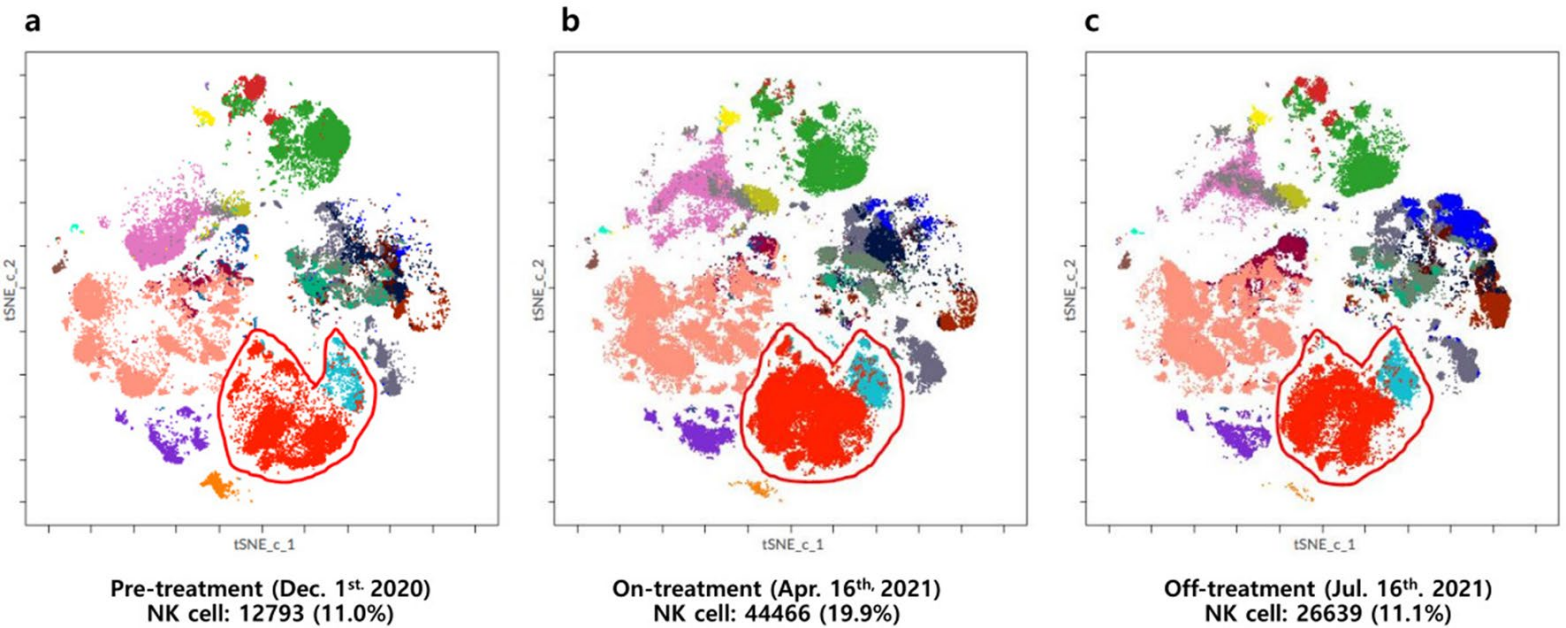
Changes in natural killer (NK) cell proportion through t-Stochastic Neighbor Embedding (t-SNE) plotting of data from patient No. 2. The orange-to-red color represents NK cells. (**A**) Pre-treatment. (**B**) On-treatment. (**C**) Off-treatment.

Figure 2 shows the NK cell changes in 6 patients with either pre-treatment or off-treatment samples. The trend of NK cell changes with lenvatinib treatment was observed in all 6 patients; the proportion of NK cells increased from baseline after treatment and decreased during the drug holiday period. The NK cell changes of pre-treatment, on-treatment, and off-treatment samples from patient No. 2 were also observed through t-SNE plotting (Fig. 3). The NK cell proportion increased from 11.0% at baseline (Fig. 3A) to 19.9% (Fig. 3B) after lenvatinib treatment, while they decreased to 11.1% (Fig. 3C) after discontinuation of lenvatinib for 7 days.

### Cytokine analysis

Next, the levels of cytokines associated with NK cell proliferation were analyzed (Table 3). IL-2 and IL-15 were NK cell activators, and IL-10 and TGFβ were NK cell inhibitors^17^. The coefficient of variation ranged from 1.7 to 20.7% in IL-2, 0.6 to 15.8% in IL-10, 1.8 to 18.2% in IL-15, and 0.2 to 17.3% in TGFβ. However, there were no significant changes in any of these cytokines due to treatment. When we compared the level of cytokines in two patients with both pre- and off-treatment samples (Patient No. 1 and 2, Supplementary Fig. 1), there were no significant changes.

**Table 3:** Cytokine analysis according to lenvatinib treatment.

	Mean concentration of cytokines (pg/ml)			*P* value^a^	*P* value^b^
	Pre-treatment (4 samples from 4 patients)	On-treatment (23 samples from 10 patients)	Off-treatment (4 samples from 4 patients)		
IL-2	0.25 (0.27)	0.09 (0.11)	0.14 (0.05)	0.31	0.37
IL-10	8.08 (3.07)	8.34 (1.81)	7.24 (1.21)	0.88	0.18
IL-15	2.50 (0.44)	3.14 (1.21)	3.67 (0.83)	0.08	0.32
TGF-beta	141.00 (7.12)	135.61 (12.43)	139.00 (6.68)	0.26	0.45

Variables are presented as means (standard deviations).

^a^*P* values for the comparison between the pre-treatment (4 samples from 4 patients) and on-treatment (23 samples from 10 patients).

^b^*P* values for the comparison between the on-treatment (23 samples from 10 patients) and off-treatment (4 samples from 4 patients).

## Discussion

This prospective study evaluated the changes in PBMCs during lenvatinib treatment in patients with advanced thyroid cancer by repeated blood sampling. Comparing the PBMC profiles during lenvatinib treatment, NK cells significantly increased with lenvatinib treatment and decreased with the discontinuation of lenvatinib. Our results present the immune-modulatory effect of lenvatinib in a real-world clinical setting.

The role of lenvatinib in the immune system was first suggested in the Hepa1-6 hepatocellular carcinoma (HCC) mouse model^11^. Antitumor activities of lenvatinib and sorafenib were not different in immunodeficient mice, but lenvatinib was more potent in immunocompetent mice. Furthermore, the antitumor activity of lenvatinib was greater in immunocompetent mice than that in immunodeficient^2,11^. Thereby, these studies suggest the immune-modulatory activity of lenvatinib in addition to its antiangiogenetic and antiproliferative effects. Zhang et al. also reported that lenvatinib inhibited murine melanoma and renal cancer, and this was associated with enhanced tumor infiltration and activation of NK cells^12^. The present study also found that lenvatinib significantly increased the proportion of NK cells in peripheral blood. In contrast, a previous study reported that sorafenib significantly reduced the number of NK cells in spleen and peripheral blood in a mice HCC model^18^. Furthermore, sorafenib inhibited reactivity of NK cells against tumor cells, thus resulting the host more susceptive to tumor growth and metastasis^18^. The difference in the immune-modulatory effect of two tyrosine kinase inhibitors on NK cells possibly contributes to the better efficacy of lenvatinib compared to that of sorafenib.

Some previous studies also evaluated peripheral blood changes after lenvatinib treatment in thyroid cancer patients^16,19^. A retrospective analysis of Phase III clinical trial of lenvatinib in DTC patients reported that a baseline neutrophil–lymphocyte ratio (NLR < 3) was associated better clinical outcomes^16^. Fukuda et al. also demonstrated that NLR values significantly decreased when the patients achieved partial response and increased upon disease progression^19^. As leukocytes were removed in PBMC samples, we could not evaluate NLR. We evaluated LMR in this study, but changes in LMR with the lenvatinib treatment was not sufficient to draw conclusion. Further studies including larger group of patients are needed. Lenvatnib monotherapy was also reported to significantly increase polymorphonuclear myeloid derived suppressor cells (PMN-MDSCs) in orthotopic anaplastic thyroid cancer (ATC) mouse model and in an ATC patient^20^. A representative human MDSC surface marker, CD33, was not included in our mass cytometry kit and we could not assess the changes in MDSC in this study.

There are some previous studies investigating the role of lenvatinib in NK cell population changes in mice models. Lenvatinib promoted the expression of chemokines on tumor cells and adhesion molecules in NK cells, which enhanced the tumor infiltration capacity of NK cells^12^. In addition, lenvatinib augmented the expression of natural cytotoxicity receptors on NK cells and thus, promoted the cytotoxicity of tumor-infiltrating NK cells^12^. We tried to investigate the levels of cytokines associated with NK cell proliferation. We selected IL-2 and IL-15 as NK cell activators and IL-10 and TGFβ as NK cell inhibitors with the literature review^17^. However, there were no significant changes, and we could not identify the underlying mechanism of how lenvatinib increased peripheral NK cell populations. A larger number of samples and additional cytokine analyses might be helpful in elucidating this mechanism.

NK cells have been described as critical contributors to innate immunity. NK cells elicit natural cytotoxicity toward malignant cells by inhibiting the expansion, proliferation, and migration of tumor cells^18,21^. In an anaplastic thyroid carcinoma mouse model, NK cells significantly inhibited the growth of the pulmonary metastasis, suggesting that NK cell-based immunotherapy may serve as an effective therapeutic approach for thyroid cancer^22^. However, some cancer cells can evade from immune surveillance by secreting cytokines which suppress NK cell functions or by attenuating of the expression of tumor-associated antigens^23–25^. To defeat this immune escape, chimeric antigen receptor (CAR)-modified NK cells are introduced, and CAR-NK cell therapy exhibited desired outcomes in both hematological and solid malignancies^25–29^. Considering the effect of lenvatinib on NK cells, sequence or combination therapies of lenvatinib with CAR-NK cell therapy are expected to show synergy in patients with thyroid cancer.

This study had several limitations. First, a small number of patients were enrolled, and there was no control group. We failed to enroll untreated or sorafenib-treated patients. We also failed to enroll patients showing disease progression during lenvatinib treatment, as a result, we were unable to evaluate NK cell changes according to disease response. Second, it is still unclear whether PBMC profiles are correlated with tumor immune cell infiltration. A future study that evaluates tumor-infiltrating immune cells in tissue specimens is warranted. Lastly, this study could not unveil how lenvatinib increases the NK cell population. With small samples, cytokine analysis did not show consistency with the immune cell population analysis. Despite these limitations, to our knowledge, this is the first study to show immune cell changes in patients with advanced thyroid cancer treated with lenvatinib.

In conclusion, there was a significant increase in the peripheral NK cell population with lenvatinib treatment in patients with advanced thyroid cancer. Our prospective clinical study confirmed that lenvatinib has an immune-modulatory effect that might be associated with lenvatinib efficacy.

### Supplementary Information

Supplementary Information.

## Data Availability

Some data sets generated during and/or analyzed during the present study are not publicly available but re available from the corresponding author on reasonable request.
