# Supplementary material for: Changes in peripheral blood immune cell population in thyroid cancer patients treated with lenvatinib
Source: Sci Rep. 2023 Aug 7;13:12765. doi: 10.1038/s41598-023-39503-w (PMC10406916; doi:10.1038/s41598-023-39503-w)

**Supplementary Table 1. Maxpar Direct Immune Profiling Assay Panel**

| **Antibody (clone)** | **Mass** |
| --- | --- |
| CD45 (HI30) | 89Y |
| CD196/CCR6 (G034E3) | 141Pr |
| CD123 (6H6) | 143Nd |
| CD19 (HIB19) | 144Nd |
| CD4 (RPA-T4) | 145Nd |
| CD8a (RPA-T8) | 146Nd |
| CD11c (Bu15) | 147Sm |
| CD16 (3G8) | 148Nd |
| CD45RO (UCHL1) | 149Sm |
| CD45RA (HI100) | 150Nd |
| CD161 (HP-3G10) | 151Eu |
| CD194/CCR4 (L291H4) | 152Sm |
| CD25 (BC96) | 153Eu |
| CD27 (O323) | 154Sm |
| CD57 (HCD57) | 155Gd |
| CD183/CXCR3 (G025H7) | 156Gd |
| CD185/CXCR5 (J252D4) | 158Gd |
| CD28 (CD28.2) | 160Gd |
| CD38 (HB-7) | 161Dy |
| CD56/NCAM (NCAM16.2) | 163Dy |
| TCRgd (B1) | 164Dy |
| CD294 (BM16) | 166Er |
| CD197/CCR7 (G043H7) | 167Er |
| CD14 (63D3) | 168Er |
| CD3 (UCHT1) | 170Er |
| CD20 (2H7) | 171Yb |
| CD66b (G10F5) | 172Yb |
| HLA-DR (LN3) | 173Yb |
| IgD (IA6-2) | 174Yb |
| CD127 (A019D5) | 176Yb |
| Live/dead intercalator-103Rh | 103 Rh |

Y: yttrium, Pr: praseodymium, Nd: neodymium, Sm: samarium, Eu: europium, Gd: gadolinium, Dy: dysprosium, Er: erbium, Yb: ytterbium, Rh: rhodiu

**Supplementary Table 2. Model phenotypes of cell populations**

| **Populations** | **Model phenotypes** |
| --- | --- |
| Lymphocytes | CD3 T cells + B cells + NK cells |
| CD3 T cells | CD8 T cells + CD4 T cells + γδ T cells + MAIT/NKT cells |
| CD8 T cells | CD45+ CD66b- CD19- CD20- CD14- CD11c- CD3+ TCRγδ- CD4- CD8+ CD161lo/- |
| CD8 naïve | CD8 T cells + CCR7hi CD45RA+ CD45RO- |
| CD8 central memory | CD8 T cells + CCR7hi CD45RA- CD45RO+ |
| CD8 effector memory | CD8 T cells + CCR7lo/- CD27+ |
| CD8 terminal effector | CD8 T cells + CCR7lo/- CD27- |
| CD4 T cells | CD45+ CD66b- CD19- CD20- CD14- CD11c- CD3+ TCRγδ- CD4+ CD8- |
| CD4 naïve | CD4 T cells + CCR7hi CD45RA+ CD45RO- |
| CD4 central memory | CD4 T cells + CCR7hi CD45RA- CD45RO+ |
| CD4 effector memory | CD4 T cells + CCR7lo/- CD45RA- CD45RO+ CD27+ |
| CD4 terminal effector | CD4 T cells + CCR7lo/- CD45RA- CD45RO+ CD27- |
| Tregs | CD4 T cells + CCR4+ CD45RA- CD45RO+ CD25hi CD127lo/- |
| Th1-like | CD4 T cells + CCR4- CD45RA- CD45RO+ CXCR3+ CCR6- |
| Th2-like | CD4 T cells + CXCR5- CD45RA- CCR4+ CXCR3- CCR6- |
| Th17-like | CD4 T cells + CXCR5- CD45RA- CCR4+ CXCR3- CCR6+ |
| γδ T cells | CD45+ CD66b- CD19- CD20- CD14- CD11c- CD45+ CD3+ CD4- CD8- TCRγδ+ |
| MAIT/NKT cells | CD45+ CD66b- CD19- CD20- CD14- CD11c- CD3+ TCRγδ- CD4- CD28+ CD161hi |
| B cells | CD45+ CD66b- CD56- CD14- CD19+ CD3- |
| B naïve | B cells + CD27- |
| B memory | B cells + CD27+ |
| Plasmablasts | B cells + CD27+ CD38+ CD20- |
| NK cells | CD45+ CD66b- CD19- CD20- CD3- CD14- CD45RA+ CD123- CD56 dim,+ |
| NK early | NK cells + CD57- |
| NK late | NK cells + CD57+ |
| Monocytes | CD45+ CD66b- CD19- CD20- CD3- CD56- CD11c+ HLA-DR+ CD14+/- |
| Monocytes classical | Monocytes + CD14+ CD38+ |
| Monocytes transitional | Monocytes + CD14 int CD38 lo/- |
| Monocytes non-classical | Monocytes + CD14- CD38- |
| DCs | CD45+ CD66b- CD19- CD20- CD3- CD14- HLA-DR+ |
| pDCs | DCs + CD123+ CD11c- |
| mDCs | DCs + CD123- CD11c+ CD38 dim,+ |
| Granulocytes | CD45 lo CD66b+ |
| Neutrophils | Granulocytes + CD294- CD16+ |
| Basophils | CD45+ CD66b- CD19- CD20- CD3- CD56- HLA-DR- CD11c- CD123+ CD294+ |
| Eosinophils | Granulocytes + CD294+ CD16- |

**Supplementary Table 3. NK cell changes according to lenvatinib treatment**

|  | **Total** | **Pre-treatment**  **(4 samples from**  **4 patients)** | **On-treatment**  **(23 samples from**  **10 patients)** | **Off-treatment**  **(4 samples from**  **4 patients)** | ***P*-value^a^** | ***P*-value^b^** |
| --- | --- | --- | --- | --- | --- | --- |
| **Absolute NK cell counts** |  |  |  |  |  |  |
| **Total NK cells** | 38550±17913 | 12465±5631 | 44202±16161 | 32134 ±7397 | <0.001 | 0.13 |
| **Early NK cells** | 5804±2162 | 2362±1344 | 6617±1689 | 4573±1063 | <0.001 | 0.02 |
| **Late NK cells** | 32745±16489 | 10103±5513 | 37585±15423 | 27561±6715 | 0.001 | 0.19 |
| **Total NK cells/CD45+ cells (%)** | 17.2±7.9 | 9.7±4.4 | 19.2±7.9 | 13.0±3.7 | 0.02 | 0.12 |

Variables are presented as means (standard deviations)

^a^ *P*-values for the comparison between the pre-treatment and on-treatment

^b^ *P*-values for the comparison between the on-treatment and off-treatment

**Supplementary Figure 1. Cytokine changes according to lenvatinib treatment**


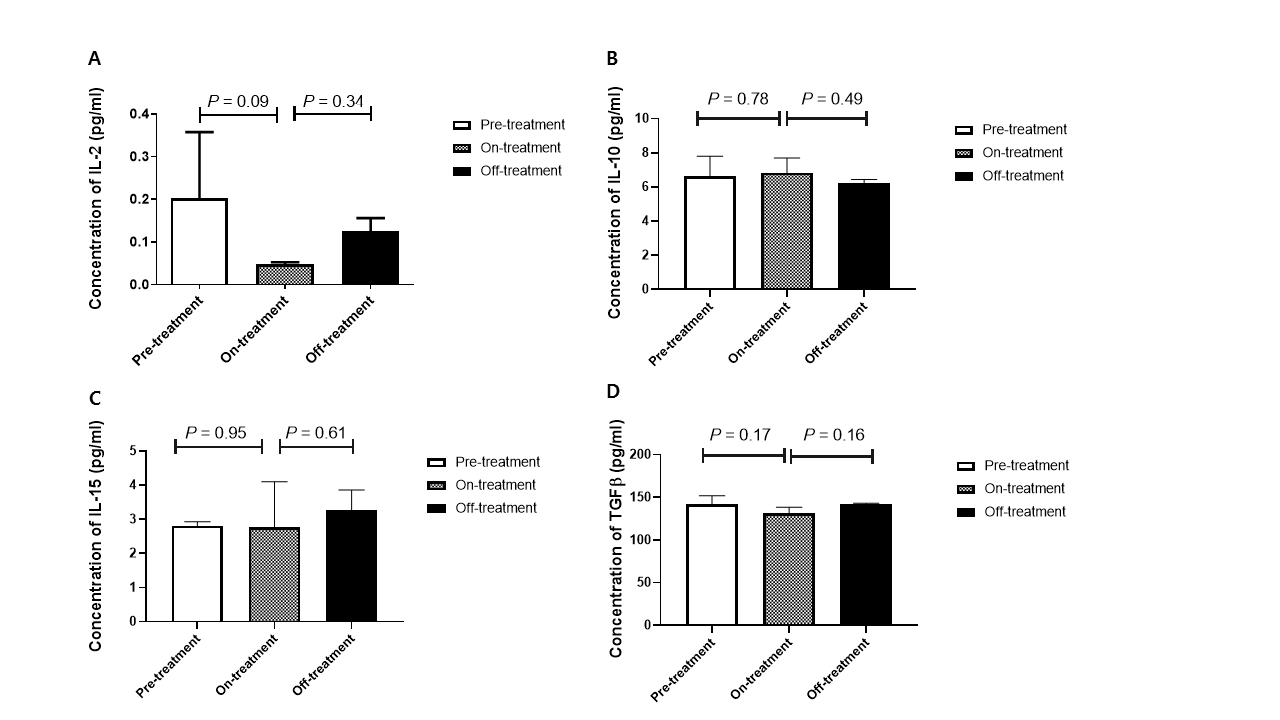

Supplement: Supplementary file 1 — Supplementary Information. [file 41598_2023_39503_MOESM1_ESM.docx]
